# Effects of Preservative Agents on Quality Attributes of Dry-Cured Fermented Sausages

**DOI:** 10.3390/foods9101505

**Published:** 2020-10-21

**Authors:** Micaela Álvarez, María J. Andrade, Carmen García, Juan J. Rondán, Félix Núñez

**Affiliations:** 1 Food Hygiene and Safety, Meat and Meat Products Research Institute, Faculty of Veterinary Science, University of Extremadura, Avda. de las Ciencias, s/n, 10003 Cáceres, Spain; maalvarezr@unex.es (M.Á.); jjrondanr@unex.es (J.J.R.); fnunez@unex.es (F.N.); 2 Food Technology, Meat and Meat Products Research Institute, Faculty of Veterinary Science, University of Extremadura, Avda. de las Ciencias, s/n, 10003 Cáceres, Spain; cgarciag@unex.es

**Keywords:** biocontrol, dry-cured fermented sausages, mycotoxins, microbial population, physio-chemical parameters, sensorial evaluation

## Abstract

*Enterococcus faecium* SE920, *Debaryomyces hansenii* FHSCC 253H, *Penicillium chrysogenum* CECT 20922, producer of the antifungal protein PgAFP, and this protein itself have previously been proposed to control toxigenic molds in dry-cured meat products. However, their effects on the usual microbial population, and the sensory characteristics of these foods, have not yet been evaluated. The aim of this study was to assess the viability of the inoculation of these protective cultures, and their impact on the quality of dry-cured fermented sausages. These microorganisms were co-inoculated with a native desirable population (*Penicillium nalgiovense*, *P. chrysogenum*, *D. hansenii*, and *Staphylococcus vitulinus*) in a dry-cured fermented sausage (salchichón)-based medium in the presence and absence of PgAFP. Macroscopically, the biocontrol candidates did not produce relevant changes in the growth of the native population, enabling their coexistence. However, PgAFP causes the alteration of the hyphae structure in desirable molds. Thus, PgAFP was discarded for use on the surface of raw dry-cured fermented sausages (salchichón) in the pilot plant. The used biocontrol agents did not negatively affect the physico-chemical parameters of the dry-cured fermented sausages (salchichón) after ripening, which showed the typical volatile profile and odor. Thus, the application of *E. faecium* SE920, *D. hansenii* FHSCC 253H, and *P. chrysogenum* CECT 20922 as protective cultures against toxigenic molds during the ripening of dry-cured fermented sausages does not modify their typical sensorial quality.

## 1. Introduction

Traditional dry-cured fermented sausages, such as “salchichón”, are highly appreciated in the Southern European countries because of their sensory characteristics. Some of these attributes are linked to the activity of the microbial population, mainly lactic acid bacteria (LAB), gram positive catalase positive cocci (GCC+), yeasts, and molds [1,2,3]. However, other microorganisms with interest from a food safety point of view could grow during the production of dry-cured fermented sausages. One of the main safety issues consists of the uncontrolled growth of toxigenic molds on the product surface, with the consequent hazard associated with the mycotoxin presence. The most frequent mycotoxin reported on dry-cured meat products is the ochratoxin A (OTA), but aflatoxins, citrinin, and cyclopiazonic acid have also been found in these foods [4,5,6,7]. Therefore, the dry-cured fermented sausage manufacturers need to implement preventive measures for reducing the presence of the previously mentioned mycotoxins. Several physical and chemical methods, with varying effectiveness, have been proposed for this purpose, but they may interfere with the appropriate ripening of these dry-cured meat products [8]. Moreover, consumers are increasingly demanding natural and additive-free products, which is considered a “decisive buying incentive” due to the common association of this term with healthier food [9]. This trend has involved searching for new alternative measures of natural origin to counteract mycotoxin production, such as biocontrol strategies. These preventive methods are based on the use of spices [10,11], or the inoculation of protective cultures, like *Enterococcus faecium*, *Debaryomyces hansenii*, and *Penicillium chrysogenum* CECT 20922 (producer of the antifungal protein PgAFP) [12,13]. These microorganisms and the protein PgAFP have shown their ability for implantation and their effectiveness against toxigenic molds in dry-cured fermented sausages. *E. faecium* isolated from Iberian dry-cured fermented sausages has demonstrated its antifungal impact on *P. nordicum*, reducing its OTA production [12]. The antagonistic effect of *D. hansenii* on *Aspergillus parasiticus* and *P. nordicum* has been reported, with a significant reduction of mycotoxins in dry-cured meat products [13,14]. Other studies have shown that *P. chrysogenum*, as well as its antifungal protein PgAFP, affected different toxigenic molds, including *Penicillium griseofulvum* and *Aspergillus flavus*, on dry-cured fermented sausages [15,16]. However, the presence of protective cultures could interfere with the beneficial microbial population and, consequently, cause undesirable changes in the technological and sensorial characteristics of dry-cured fermented sausages. To the best of our knowledge, this topic has not been studied yet, despite the fact that the physico-chemical, textural, and sensorial characteristics of foods are crucial aspects for consumer acceptance [17,18]. As stated above, some typical characteristics of dry-cured fermented sausages are related to the native population, or the starter cultures added to provide a standardized flavor [19]. The action of LAB is essential to decrease the pH of the product, producing lactic acid by fermentation and, consequently, limiting the development of pathogenic and spoilage microorganisms [20]. In addition, some of the LAB suggested as starter cultures have shown a probiotic effect, providing a benefit to human health [21]. The group of GCC+ contributes to the development of the appropriate color and flavor [20]. Yeasts and molds make a huge contribution to the peculiar flavor that characterizes dry-cured meat products [1,2,22].

Therefore, before using microorganisms for controlling toxigenic molds in dry-cured fermented sausages, it is necessary to assess their effects on both the usual microbial population, and the sensory characteristics of such products. Additionally, the potential antagonism between biocontrol agents and the native population of sausages should be checked by studying their growth in dual cultures on a meat substrate [23].

The objective of this study was to test the interactions between potential protective cultures and the native population of dry-cured fermented sausages. This study is the first approach to evaluating the effects of the previously proposed biocontrol candidates on the sensorial quality of dry-cured fermented sausages, by studying the changes of their physico-chemical, textural, and sensorial parameters.

## 2. Materials and Methods

### 2.1. Microorganisms

Three previously suggested biocontrol agents isolated from dry-cured meat products were tested: *P. chrysogenum* CECT 20922 (Pc), producer of the antifungal protein PgAFP [16], from the Spanish Type Culture Collection (CECT; Valencia, Spain); *D. hansenii* FHSCC 253H (Dh) from the Culture Collection of the Food Hygiene and Safety Research Group of the University of Extremadura (FHSCC; Cáceres, Spain) [14], and *E. faecium* SE920 (Ef) from the Food Quality and Microbiology Research Group of the University of Extremadura (Badajoz, Spain) [24]. The native desirable microorganisms isolated from dry-cured meat products *Penicillium nalgiovense* FHSCC Pj261 (Pn), *P. chrysogenum* FHSCC Pg222 (Pg222), *D. hansenii* FHSCC 46P (Dh46P), and *Staphylococcus vitulinus* FHSCC MSA19 (Sv) belonged to the FSHCC.

### 2.2. Inocula Setting

The mold inocula were prepared by growing on potato dextrose agar (PDA; Scharlab, S.L.; Barcelona, Spain) at 25 °C for 7 days. Conidia were harvested by washing the surface of the plates with 3 mL of phosphate-buffered saline (PBS) containing 0.32 g/L NaH_2_PO_4_ (Scharlab, S.L.), 1.09 g/L Na_2_HPO_4_ (Scharlab, S.L.), and 9 g/L of NaCl (Fisher Scientific S.L.; Waltham, MA, USA). Each conidia suspension was quantified using a Thoma counting chamber (Blaubrand^®^; Wertheim, Germany) and adjusted to 10^5^ spores/mL to be used as inoculum.

*D. hansenii* strains were incubated in yeast extract-sucrose broth (YES; 20 g/L yeast extract (Scharlab, S.L.)) and 125 g/L sucrose (Scharlab, S.L.) at 25 °C for 24 h under stirring conditions (150 rpm). After centrifuging, the pellet was resuspended in PBS and quantified using the Thoma counting chamber before adjusting to 10^6^ cfu/mL.

*E. faecium* and *S. vitulinus* were cultured in brain heart infusion broth (BHI, Scharlab, S.L.) and incubated for 48 h at 30 °C under stirring (150 rpm). After their centrifugation, the pellets were resuspended in PBS and turbidimetrically adjusted to 10^6^ cfu/mL.

The protein PgAFP was extracted by fast protein liquid chromatography (FPLC) using the method previously described by Delgado et al. [16].

### 2.3. Experimental Design

#### 2.3.1. Dual-Culture Assay

Each biocontrol agent was co-inoculated with each native microorganism in a dual-culture assay, based on Magan and Lacey [23], with some modifications [25]. The culture medium was prepared with lyophilized Spanish dry-cured fermented sausages (salchichón) (3% *w*/*v*) and 20 g/L of bacteriological agar (Scharlab, S.L.). In addition, 5% and 9% (*w*/*v*) of NaCl was added to achieve water activity values of 0.97 (*a*_w_; DFS-0.97) and 0.94 *a*_w_ (DFS-0.94), respectively. The *a*_w_ was determined using a LabMaster *a*_w_ meter (Novasina AG; Lachen, Switzerland). The effect of the antifungal protein PgAFP on the native positive population was also tested by adding 20 µg/mL to each culture media (DFS-0.97P and DFS-0.94P).

The plates were incubated for 7 days at three different temperatures: 25, 20, and 15 °C. The interactions between microorganisms were macroscopically and microscopically visualized using a microscope, NIKON Eclipse E200 (NIKON; Tokyo, Japan). The sampling of mycelia was performing by scraping them with a sterile scalpel immediately before visualizing under the microscope.

#### 2.3.2. Pilot Plant Assay

Raw sausages made with pork meat and backfat, salt, garlic, sugar, dextrose, and black pepper were purchased from a local industry and inoculated on their surface with different combinations of the protective cultures. Five different batches were prepared: a non-inoculated control (C), one inoculated with *E. faecium* SE920 (E), one inoculated with *D. hansenii* FSHCC 253H (D), one inoculated with *P. chrysogenum* CECT 20922 (P), and one inoculated with a mix of the three biocontrol agents (M). Each batch included 5 replicates. The sausages were ripened in pilot plants for 21 days adjusting the relative humidity (RH) and temperature to the natural process in the meat industry. The cycle of maturation started with 3 days at 4 °C and 85% RH, followed by 1 day at 13 °C and 84% RH, and, finally, 17 days at 12 °C and 84% RH.

### 2.4. Physico-Chemical Analysis

The pH, *a*_w_, and moisture were measured at the end of the ripening to assure the correct processing of the sausages. The pH of the dry-cured fermented sausages was evaluated using a pH meter electrode, model FC232D (HANNA Instruments S.L.; Eibar, Spain). The *a*_w_ was measured as previously mentioned. The moisture was analyzed following the reference method 935.29 from the AOAC [26], drying at 105 °C.

### 2.5. Instrumental Texture

The texture profile analysis (TPA) was performed in slices 1 cm thick using a TA.XT plus Texture Analyser (Stable Micro Systems Ltd.; Godalming, UK). The samples were axially compressed to 50% at 2 mm/s, with a 2-cycle sequence using a flat plunger of 50 mm in diameter (P/50). The texture parameter values were obtained from the force deformation curves, previously described by Bourne [27], and the hardness, adhesiveness, springiness, cohesiveness, and chewiness were analyzed.

### 2.6. Instrumental Colour

The color was measured in slices 1 cm thick with a Minolta CR-300 colorimeter (Konica Minolta, Inc.; Nieuwegein, The Netherlands) using the CIE L*a*b* color space determined by luminosity (L*), redness (a*), and yellowness (b*). The display area was 2.5 cm, with an illuminant D65, and an observer angle of 0°. All samples were measured by triplicate.

### 2.7. Volatile Compounds Analysis

The volatile compounds were extracted by solid phase microextraction (SPME) after heating to 37 °C for 30 min, using a divinylbenzene-carboxen-polydimethylsiloxane (DVB/CAR/PDMS) 50/30 µm fiber (Merck; Darmstadt, Germany). They were then analyzed by gas chromatography-mass spectrometry (GC-MS) in a gas chromatograph 6890 GC (Agilent Technologies; Santa Clara, CA, USA) equipped with a HP-5 column (5% phenyl−95% dimethylpolysiloxane) and coupled to a mass spectrometer detector, 5975C (Agilent Technologies). Oven temperature started at 40 °C for 5 min and was increased to 280 °C, with a rate of 7 °C/min. The desorption time was 30 min at 250 °C. The transfer line temperature was established at 280 °C. The carrier gas was helium with a flow rate of 1.2 mL/min. MS detection was performed in full scan (50–350 amu). Automated peak find and spectral deconvolution were used for data treatment, and the dentification of the volatile compounds was achieved by comparing their mass spectra with the NIST/EPA/NIH library.

### 2.8. Sensory Evaluation

The sensory evaluation consisted of a hedonic preference test and an ordination test using a scale of odor intensity. The analysis was carried out by untrained volunteers (22) recruited at the Faculty of Veterinary Sciences of the University of Extremadura. Each sample was presented to the panelists labelled with a three-digit random codes, and served at room temperature in covered Falcon tubes to avoid visual influences. For the hedonic test, the panelists had to order the samples corresponding to each batch from the least preferred to the most preferred. For the scale of odor intensity, the panelists had to order the samples corresponding to each batch from the least intensity to the most intensity. Re-tasting of samples was permitted to confirm the rankings. The results were analyzed through rank sums scores following the methodology described by Hein et al. [28]. Thus, the scores show the aggregate of the positions in the scale for each sample, the highest results being the most preferred or intense. Subsequently, a slice of each batch was presented to the panelists and they were asked if they would purchase it or not.

### 2.9. Statistical Analysis

The statistical analysis was performed using SPSS IBM v.22 software (IBM; New York, NY, USA). The non-parametric Kruskal–Wallis and Mann–Whitney tests were used, since the data failed the normality and homoscedasticity tests. The analysis of correlations between data from the parameters of color was carried out using the Spearman correlation test. Friedman’s chi-square test was applied to the sensorial test results. The statistical significance was stablished at *p* ≤ 0.05.

## 3. Results and Discussion

Since microorganisms have a great impact on the development of the sensory characteristics of dry-cured fermented sausages, the study of the interaction between the candidates for biocontrol agents and the beneficial population of this product is a key issue to avoid disturbance in the microbial population during the ripening and, consequently, in the quality of the final product [29].

### 3.1. In Vitro Effect of the Biocontrol Agents on the Growth of Beneficial Microorganisms

**Figure 1:**
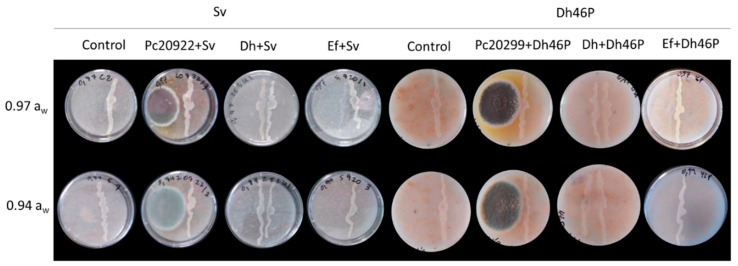
Interactions of three biocontrol agents (*Enterococcus faecium* SE920, *Debaryomyces hansenii* FSHCC 253H, and *Penicillium chrysogenum* CECT 20922) with native dry-cured meat product microorganisms (*Staphylococcus vitulinus* MSA19 and *D. hansenii* 46P) at 0.97 and 0.94 water activity (*a*_w_) after 7 days at 25 °C in a dry-cured fermented sausage (salchichón)-based medium. Treatments: Control: native microorganism inoculated without biocontrol agents; Sv: *S. vitulinus* MSA19; Dh46P: *D. hansenii* 46P; Pc20299: *P. chrysogenum* CECT 20922; Dh: *D. hansenii* FSHCC 253H; Ef: *E. faecium* SE920. The biocontrol agent was inoculated on the left side of the plate and the tested microorganism on the right side of the plate.

**Figure 2:**
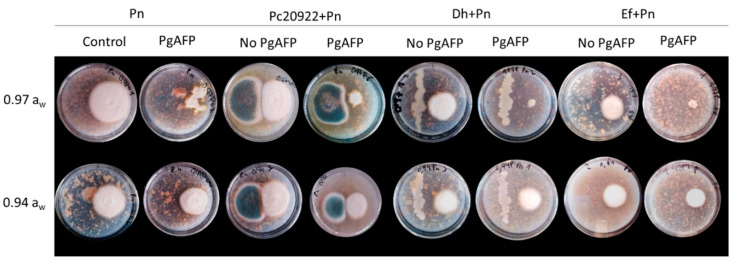
Interactions of the three biocontrol agents (*Enterococcus faecium* SE920, *Debaryomyces hansenii* FSHCC 253H, and *Penicillium chrysogenum* CECT 20922) with the native dry-cured meat product mold *Penicillium nalgiovense* FSHCC Pj261 at 0.97 and 0.94 water activity (*a*_w_) after 7 days at 25 °C in the presence and absence of the antifungal protein PgAFP in a dry-cured fermented sausage (salchichón)-based medium. Treatments: Control: *P. nalgiovense* FSHCC Pj261 inoculated without biocontrol agents; Pn: *P. nalgiovense* FSHCC Pj261; Pc20922: *P. chrysogenum* CECT 20922; Dh: *D. hansenii* FSHCC 253H; Ef: *E. faecium* SE920. No PgAFP: PgAFP was not added; PgAFP: PgAFP was added. The biocontrol agent was inoculated on the left side of the plate and *P. nalgiovense* FSHCC Pj261 on the right side of the plate.

**Figure 3:**
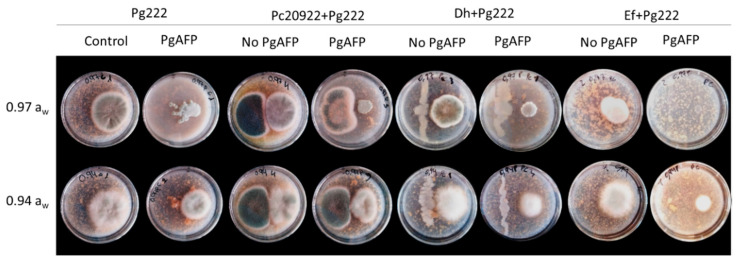
Interactions of the three biocontrol agents (*Enterococcus faecium* SE920, *Debaryomyces hansenii* FSHCC 253H, and *Penicillium chrysogenum* CECT 20922) with native dry-cured meat product mold *P. chrysogenum* FSHCC Pg222 at 0.97 and 0.94 water activity (*a*_w_) after 7 days at 25 °C in the presence and absence of the antifungal protein PgAFP in dry-cured fermented sausage (salchichón)-based medium. Treatments: Control: *P. chrysogenum* FSHCC Pg222 inoculated without biocontrol agents; Pg222: *P. chysogenum* FSHCC Pg222; Pc20922: *P. chrysogenum* CECT 20922; Dh: *D. hansenii* FSHCC 253H; Ef: *E. faecium* SE920. No PgAFP: PgAFP was not added; PgAFP: PgAFP was added. The biocontrol agent was inoculated on the left side of the plate and *P. chrysogenum* FSHCC Pg222 on the right side of the plate.

The biocontrol agents Ef, Dh, and Pc did not affect the growth of Sv nor Dh46P in dual cultures in the four media (DFS-0.97, DFS-0.94, DFS-0.97P, and DFS-0.94P) at every combination of *a*_w_ and temperature tested. In Figure 1, co-inoculated plates of Sv and Dh46P with the biocontrol agents incubated at 25 °C are shown as an example. The diameter of the colonies of Pn (Figure 2) and Pg222 (Figure 3) was slightly lower in the presence of the biocontrol agents than in their absence, but not completely impaired. Regarding the co-inoculation with Pc, mutual inhibition at contact was observed for both beneficial molds in DFS-0.97 and DFS-0.94 (Figure 2 and Figure 3).

The marginal impact found could be attributed to the different mechanism of action reported for the studied biocontrol agents. Thus, the antagonistic effect of *D. hansenii* Dh253H by nutritional competition, and the production of extracellular soluble and volatile antifungal compounds have previously been described [30]. The protective *P. chrysogenum* CECT 20922 antagonism against Pg222 and Pn could be related to competition for nutrients and space, as previously described against *P. nordicum* [25]. However, this limited action of the protective cultures on the growth of both non-toxigenic molds did not completely inhibit their growth. Thus, they could coexist without affecting their contribution to the development of the sensory characteristics of sausages. The three biocontrol agents were then used for manufacturing dry-cured fermented sausages in the pilot plant assay.

**Figure 4:**
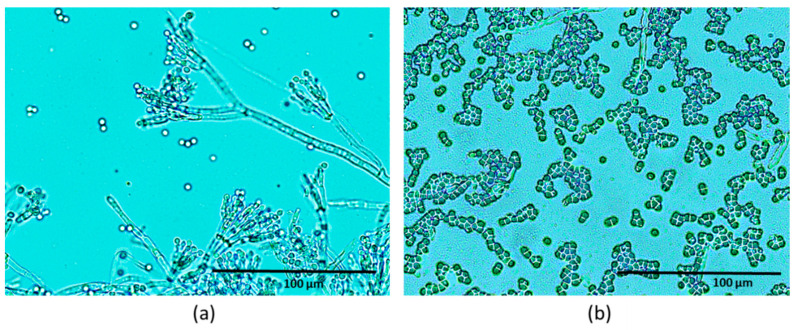
Microscopical image of *Penicillium nalgiovense* Pj261 in the absence (**a**) and presence (**b**) of the antifungal protein PgAFP after 15 days of incubation at 25 °C and 0.97 water activity in a dry-cured fermented (salchichón)-based medium.

On the other hand, the presence of the antifungal protein PgAFP did not affect the growth of Sv nor Dh46P. However, the protein caused a decrease in the growth of Pn and Pg222, mainly at 0.97 *a*_w_ (Figure 2 and Figure 3, respectively). In addition, at this *a*_w_ value, the colonies of Pn had an abnormal appearance in all conditions tested. The colonies lost their circular shape and seemed to be composed by multiple dots. When visualized under microscopy, the mycelia were brittle, not presenting the characteristic structure of the hyphae (Figure 4).

These results are consistent with the antifungal effect of PgAFP, mainly related to the repression of the cell wall integrity pathway (CWI) and chitin biosynthesis in sensitive molds [31], which could entail a reduction of the aerial mycelium [32]. As a consequence, PgAFP should not be recommended to be added to the surface of dry-cured fermented sausages where desirable molds are expected to grow. Thus, PgAFP was not selected for the following experiment in the pilot plant.

### 3.2. Effect of Biocontrol Agents on the Dry-Cured Fermented Sausages Characteristics

**Figure 5:**
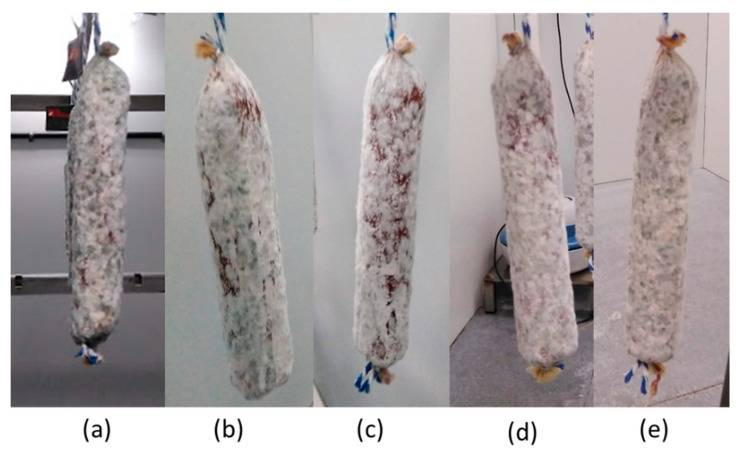
External appearance of dry-cured fermented sausages throughout ripening. (**a**): batch C (without biocontrol agents); (**b**): batch E (*Enterococcus faecium* SE920); (**c**): batch D (*Debaryomyces hansenii* FHSCC 253H); (**d**): batch P (*Penicillium chrysogenum* CECT 20922); (**e**): batch M (*E. faecium* SE920 + *D. hansenii* FHSCC 253H + *P. chrysogenum* CECT 20922).

Sausages inoculated with different combinations of the biocontrol agents were then ripened (Figure 5) to assess the influence of these microorganisms on the physico-chemical and sensory characteristics of the final products. After the inoculation of Ef, Dh, and Pc on the surface of sausages, and their drying and ripening, the physico-chemical parameters were measured (Table 1).

The biocontrol agent Pc grew properly in the inoculated batches P and M (Figure 5). Nonetheless, the surfaces of the sausages from the remaining batches were gradually covered by spontaneous molds from the native population during the ripening period (Figure 5), as happens in the traditional industries.

The pH of the sausages was not significantly affected by the inoculation of Pc and the mix of the biocontrol agents when compared with the non-inoculated batch (Batch C; Table 1). However, the use of Ef and Dh slightly changed the pH (batches E and D, respectively). It is well known that LAB acidify dry-cured fermented sausages throughout the fermentation stage, with the subsequent improvement of their safety [33]. On the contrary, Dh causes a small rise in the pH, that could be a consequence of the increased proteolytic activity or the lactic acid consumption [18,34].

The *a*_w_ in all the batches inoculated with the biocontrol agents was significantly lower with respect to batch C (Table 1). However, no differences were observed in the moisture among batches (Table 1). That is in accordance with other studies performed on dry-cured fermented sausages with the addition of *D. hansenii* [34], because it favors dehydration through proteolysis; and LAB are linked to the decrease in the water-holding capacity of denaturalized proteins, in the last case induced by the acidification [35]. The reduction of *a*_w_ in batch P could be related to the capacity for holding water by molds growing on the surface of dry-cured fermented sausages [36]. The mix of the three biocontrol agents was also significantly different with respect to the control, showing the lowest *a*_w_ value (0.85). However, no differences among batches were detected in the moisture that could indicate a uniform drying process.

Despite the fact that the inoculated batches showed some differences in physico-chemical parameters with respect to the control, the obtained values were within the usual range for dry-cured fermented (salchichón), the pH being from 5 to 6 [3,37], *a*_w_ from 0.80 to 0.87 [18,38], and moisture from 20% to 44% [3,39]. Therefore, the studied protective cultures did not lead to relevant disturbances of these parameters through the ripening process of the sausages.

Regarding the instrumental texture parameters, similar results were obtained in the five manufactured batches, their values being in the common ranges of dry-cured fermented sausages: for hardness, from 74.4 to 269.2 N [17,40]; springiness, from 0.55 to 0.8 [41,42]; cohesiveness, from 0.39 to 0.8 [3,17]; and chewiness, from 94.8 to 152.4 N [3]. However, all the sausages showed higher levels of adhesiveness than others previously reported, from −0.44 to −2.44 [17,42]. This difference could be due to a high fat content [17] that can achieve the 60% of dry matter. Significant differences (*p* < 0.05) in the springiness and cohesiveness were only found in batch P, with respect to batch C (Table 1). The significant reductions of springiness and cohesiveness in this batch could be related to an intense proteolysis [40], due to the high production of proteases by *P. chrysogenum* [43].

Concerning the CIE L*a*b* parameters, no differences were observed, with the exception of the parameter L* in batches D and P that increased with respect to the control (Table 1). The increase in lightless by the inoculation of *D. hansenii* has also been reported in dry cured-fermented sausages [42]. For the parameters a* and b* in batch M, both values were the lowest (Table 1). When evaluating the correlation among the color parameters, a positive relation (0.854) was found between parameters a* and b* (*p* ≤ 0.01), which indicates that both reductions are likely related. The rise of a* in meat products has been related to the increase of lactic acid that interferes in the denaturation of myoglobin and moisture loss [44]. Nonetheless, in the present study the pH and moisture values did not show differences between batches C and M, so the changes in redness seem not to be linked to these parameters. The decrease in the parameter b* has been attributed to the drop of oxymyoglobin, probably due to the oxygen consumption by microorganisms [44]. Therefore, the highest microbial load inoculated in batch M, due to the co-inoculation of the three biocontrol agents, could be responsible for the reduced redness and yellowness in these sausages.

A total of 42 volatile compounds were identified and quantified in the dry-cured fermented sausages after ripening (Table 2), among them being those usually found in dry-cured fermented sausages [1,45]. The volatile pattern of the different batches was the result of the addition of spices to a complex meat matrix, combined with the reactions derived from lipid auto-oxidation and the activity of both native microorganisms and the inoculated biocontrol agents.

The results are categorized by groups according to their possible origin, such as lipid oxidation (16), carbohydrate fermentation (1), amino acid catabolism (11), and spices (14).

A total of 35 compounds were detected in batches C, D, and M, followed by batches P (34) and E (33). Batch D showed the lowest number of compounds derived from lipid oxidation, and the highest from amino acid catabolism. The unique compound coming from carbohydrate fermentation, acetoin, and all the compounds resulting from spices were detected in every batch. The volatile compounds originating from spices showed the highest amounts. Terpenes were most of the compounds derived from spices, 3-carene and D-limonene being predominant in all batches. Among them, 3-carene derived from black pepper [46] was the volatile compound showing the highest amount, since this ingredient is one of the main spices added to “salchichón” [38,41]. The heterogeneity in the distribution of spices in the matrix, mainly the whole peppercorns, could explain the differences found among the batches.

Regarding the volatile compounds from lipid oxidation, there were no substantial differences between the batches, and no large variations in flavor notes related to these compounds, such as rancidity [47], should thus be expected. Since 1-propanol and 2,3-octanedione were only found in batch E, they were likely produced by the action of *E. faecium*. Both compounds have been previously associated with enterococci metabolism in dry-cured fermented sausages [45]. The 3-octanone was also found in higher levels in batches E (0.60) and M (0.26) than in batch C (0.13), suggesting that *E. faecium* was the main producer of this ketone. This compound is responsible for a mushroom or musty aroma, and has been ascribed to the presence of other LAB, such as *Carnobacterium maltaromaticum*, in meat [48,49]. The volatiles 4-heptanone and 2-methylbutanal were only found in batches D and M. Heptanal was exclusively found in batches E and P, and nonanoic acid in batches E and D. Among ketones derived from lipid oxidation, the level of 4-heptanone was significantly higher in batch M, and 3-octanone in batch E, compared to batch C (*p* ≤ 0.05). Regarding amino acid catabolism, alcohols were mostly identified, the 3-methyl-1-butanol standing out with the highest amount in all batches. The detection of 2-methyl-1-propanol was remarkable in all inoculated batches (E, D, P, and M), but not in batch C. Moreover, batch D showed the highest amounts of 2-methyl-1-propanol, 3-methylbutanal, 2-methylbutanal, and 3-methylbutanoic acid (*p* ≤ 0.05). Some of these branched-chain volatiles, such as 3-methylbutanal, 2-methyl-1-propanol, 3-methyl-1-butanol, and 2-methylpropanoic acid, have been associated with the typical aroma of dry-cured meat products [50]. 3-methylbutanal and 3-methylbutanoic acid have been related to the activity of yeasts in meat substrates [1,51], which could clarify the highest level of these compounds being found in batch D. The limited differences found in the pattern of volatile compounds between batches is consistent with the small influence that protective cultures have shown on the growth of microorganisms usually present in dry-cured fermented sausages (Figure 1, Figure 2 and Figure 3). Therefore, although the tested microorganisms caused small changes in the generation of volatiles that contribute to the characteristic aroma of dry-cured fermented sausages in the different batches, it is not expected that such modifications can lead to significant variations in the odor of the sausages. In this sense, no significant differences were detected by the tasting panel between batches in preference ranking, intensity of odor scale, and buying preference (Table 3). Concerning the purchase preferences, 18 of 22 panelists would buy all the batches. These positive aspects support the implementation of these biocontrol agents since they did not affect the purchase intention of the panelists.

## 4. Conclusions

In conclusion, the biocontrol candidates *E. faecium* SE920, *D. hansenii* FHSCC 253H, and *P. chrysogenum* CETC 20,922 did not have a significant influence on the native population of dry-cured fermented sausages. However, the application of PgAFP on the surface of products, where desirable molds are expected to grow, could not be recommended. In addition, the superficial inoculation of these three protective microorganisms individually or combined in dry-cured fermented sausages did not significantly modify the physico-chemical parameters, nor the sensorial properties of the product. Therefore, there are no technological drawbacks against the application of these microorganisms as protective cultures for the biocontrol of toxigenic molds during the ripening of dry-cured fermented sausages.

## Figures and Tables

**Table 1:** Values of pH, water activity (*a*_w_), moisture, instrumental texture parameters (hardness, adhesiveness, springiness, cohesiveness, and chewiness), and CIE L*a*b* parameters of dry-cured fermented sausage (salchichón) inoculated with biocontrol agents after 21 days of ripening.

Parameters	Batches				
	C ^a^	E	D	P	M
pH	5.76 ± 0.01	5.62 ± 0.11 *	6.08 ± 0.08 *	5.81 ± 0.14	5.78 ± 0.06
*a*_w_	0.86 ± 0.00	0.85 ± 0.00 *	0.86 ± 0.00 *	0.86 ± 0.00 *	0.85 ± 0.00 *
Moisture (%)	37.99 ± 1.08	37.72 ± 1.09	38.00 ± 2.23	35.60 ± 2.34	37.18 ± 0.67
Hardness (N)	216.95 ± 27.50	203.49 ± 21.11	232.38 ± 9.21	211.69 ± 15.40	165.01 ± 40.15
Adhesiveness (N/s)	−9.85 ± 1.22	−11.41 ± 1.27	−11.61 ± 1.03	−10.97 ± 0.74	−10.80 ± 3.37
Springiness	0.74 ± 0.06	0.70 ± 0.09	0.80 ± 0.07	0.66 ± 0.03 *	0.75 ± 0.16
Cohesiveness	0.61 ± 0.00	0.62 ± 0.00	0.61 ± 0.00	0.59 ± 0.00 *	0.62 ± 0.00
Chewiness(N)	100.76 ± 15.84	89.94 ± 18.63	115.62 ± 10.42	82.70 ± 7.67	78.02 ± 26.50
*L* *	42.68 ± 0.31	42.33 ± 2.95	45.24 ± 1.57 *	45.38 ± 3.08 *	45.35 ± 5.21
*a* *	19.99 ± 2.39	19.21 ± 0.58	20.67 ± 1.42	18.31 ± 1.2	16.34 ± 2.0 *
*b* *	6.74 ± 0.89	5.83 ± 0.21	6.88 ± 0.92	5.85 ± 0.89	4.43 ± 0.78 *

^a^ C: non-inoculated control; E: *Enterococcus faecium* SE920; D: *Debaryomyces hansenii* FHSCC 253H; P: *Penicillium chrysogenum* CECT 20922; M: inoculated with the three biocontrol agents. * Indicates statistical differences with respect to the control (*p* ≤ 0.05).

**Table 2:** Volatile compounds ^a^ identified and quantified from dry-cured fermented sausages (salchichón) inoculated with biocontrol agents after 21 days of ripening.

Origin/Compound	Id ^b^	Batches ^c^				
		C	E	D	P	M
**Lipid oxidation**						
1-propanol	MS	n.d. ^d^	0.07 *	n.d.	n.d.	n.d
1-hexanol	MS	2.25	6.43	n.d. *	5.24	2.41
2-heptanol	MS	0.18	n.d. *	0.15	0.17	0.16
Hexanal	MS/Rf	1.63	8.25	0.45 *	3.32	1.63
Heptanal	MS/Rf	n.d.	0.71 *	n.d.	0.42 *	n.d.
Octane	MS	0.16	n.d. *	n.d. *	0.22	0.15
2-heptanone	MS	0.66	0.47	0.91	1.01	n.d. *
4-heptanone	MS	n.d.	n.d.	0.14 *	n.d.	1.52 *
2-octanone	MS	0.10	n.d. *	0.08 *	n.d. *	0.17
3-octanone	MS	0.13	0.60 *	n.d. *	0.31	0.26 *
2,3-octanedione	MS	n.d.	0.22 *	n.d.	n.d.	n.d.
Hexanoic acid	MS	0.88	2.18	0.52 *	1.10	0.40 *
Octanoic acid	MS	0.39	0.41	0.44	0.19 *	0.34
Nonanoic acid	MS	n.d.	0.28 *	0.27 *	n.d.	n.d.
1-octen-3-ol	MS	1.87	4.42	0.92 *	2.75	1.47
2-nonanone	MS	0.19	n.d. *	0.19	0.35	0.27
**Carbohydrate fermentation**						
Acetoin	MS	1.40	2.40	1.97	2.39	0.90
**Amino acid catabolism**						
2-methylpropanal	MS/Rf	0.14	n.d. *	0.08	n.d. *	n.d. *
2-methyl-1-propanol	MS	n.d.	0.17 *	0.19 *	0.17 *	0.18 *
3-methylbutanal	MS/Rf	0.14	0.22	0.34 *	0.09 *	0.23
2-methylbutanal	MS/Rf	n.d.	n.d.	0.09 *	n.d.	0.07 *
3-methyl-1-butanol	MS	1.56	1.17	2.02	1.29	1.64
2-methyl-1-butanol	MS	0.28	0.26	0.28	0.24	0.27
2-methylpropanoic acid	MS	0.34	0.29	0.40	0.16 *	0.41
2-methylbutanoic acid	MS	0.23	0.21	0.20	0.13	0.28
3-methylbutanoic acid	MS	0.46	n.d. *	0.84 *	0.29	0.49
2-ethyl-1-hexanol	MS	0.25	0.32	n.d. *	n.d. *	n.d. *
Phenylethyl alcohol	MS	0.22	n.d. *	0.25	0.22	0.20
**Spices**						
Thujene	MS	0.32	0.27	0.35 *	0.22 *	0.27
α-pinene	MS	2.25	2.40	2.60	1.69	1.67 *
β-pinene	MS	3.63	3.37	4.22	3.07	2.58 *
α-phellandrene	MS	0.57	0.41	0.60	0.40 *	0.46 *
β-phellandrene	MS	0.74	0.60	1.03	0.37 *	0.38
3-carene	MS	7.63	7.00	8.92	5.87	4.91 *
o-cymene	MS	1.03	1.61	1.06	1.06	0.93
D-limonene	MS	4.71	4.11	5.40	3.88	3.18 *
γ-terpinene	MS	0.15	0.26 *	0.21 *	0.11 *	0.10 *
L-terpinen-4-ol	MS	0.48	0.40 *	0.57 *	0.42	0.40 *
α-terpineol	MS	0.18	0.17	0.23 *	0.17	0.17
Safrole	MS	0.11	0.10 *	0.13 *	0.09 *	0.10
Caryophyllene	MS	0.77	0.77	0.98	0.65	0.68
Myristicin	MS	0.59	0.50	0.79	0.42	0.47

^a^ Results are expressed in arbitrary area units (×10^−6^), as means of 3 replicates of each batch. ^b^ Id: reliability of identification: MS: chromatogram deconvolution and identification by comparing the mass spectrum of the compounds with the NIST/EPA/NIH database; Rf: mass spectrum and retention time identical with a reference compound. ^c^ C: non-inoculated control; E: *Enterococcus faecium* SE920; D: D*ebaryomyces hansenii* FHSCC 253H; P: *Penicillium chrysogenum* CECT 20922; M: inoculated with the three biocontrol agents. ^d^ n.d.: not detected. *: Indicates statistical differences in the amount of a volatile compound, with respect to the control (*p* ≤ 0.05).

**Table 3:** Rank sums scores for preference ranking and the odor intensity scale of dry-cured fermented sausages (salchichón) inoculated with the biocontrol agents after 21 days of ripening. *p-*values from Friedman’s chi-square test are also shown.

Test	Batch ^a^	Rank Sums	*p-*Value ^b^
Preference ranking	C	52	0.329
	E	70	
	D	71	
	P	67	
	M	70	
Intensity of odor	C	69	0.656
	E	66	
	D	69	
	P	56	
	M	70	

^a^ C: non-inoculated control; E: *Enterococcus faecium* SE920; D: *Debaryomyces hansenii* FHSCC 253H; P: *Penicillium chrysogenum* CECT 20922; M: inoculated with the three biocontrol agents. ^b^ Statistical significance stablished at *p* ≤ 0.05.
